# Dissolving Microneedle Patch Incorporated with Insulin Nanoparticles for The Management of Type-I Diabetes Mellitus: Formulation Development and in Vivo Monitoring

**DOI:** 10.34172/apb.025.42583

**Published:** 2025-06-10

**Authors:** Bheemisetty Brahmam, Salwa, Dani Lakshman Yarlagadda, Prasad Chowdari Gurram, Lalit Kumar, Rekha R Shenoy, Shaila A Lewis

**Affiliations:** ^1^Department of Pharmaceutics, Manipal College of Pharmaceutical Sciences, Manipal Academy of Higher Education, Manipal– 576104, India; ^2^Department of Quality of Assurance, Manipal College of Pharmaceutical Sciences, Manipal Academy of Higher Education, Manipal– 576104, India; ^3^Department of Pharmacology, Manipal College of Pharmaceutical Sciences, Manipal Academy of Higher Education, Manipal– 576104, India; ^4^Department of Pharmaceutics, National Institute of Pharmaceutical Education and Research, Hajipur- 844102, India

**Keywords:** Microneedle, Insulin nanoparticles, Diabetes, Carboxy phenyl boronic acid, Chitosan conjugate

## Abstract

**Purpose:**

The present study aimed to fabricate microneedles (MNs) for transdermal delivery of insulin. Chitosan-conjugated carboxy phenyl boronic acid polymer was synthesized and characterized to load insulin in the form of nanoparticles.

**Methods:**

Optimized insulin nanoparticles (ILN-NPs) were loaded into MN arrays by micromolding, and the resulting MN patches were characterized by scanning electron microscopy (SEM) and mechanical failure tests. The MNs were evaluated for skin insertion via a confocal laser scanning microscope. The in vivo efficacy (blood glucose levels [BGLs] and serum insulin concentration) of the MNs was studied in diabetic rats in comparison with traditional subcutaneous insulin injection.

**Results:**

In diabetic rats treated with MNs incorporated with insulin-loaded nanoparticles (ILN-MNs), the BGLs reached≤200 mg/dL at 2 h following the application of the ILN-MNs and maintained BGLs≤200 mg/dL from 2-8 h. The BGLs decreased to 29 mg/dL at 2 h following the subcutaneous administration of insulin. After 6 h, the BGLs rose to their initial level. These results were supported by the corresponding serum insulin concentrations.

**Conclusion:**

The findings of this study demonstrate the reliability of the developed ILN-MNs for sustaining normal BGLs in diabetic rats. Therefore, it can be further explored as an approach for diabetes treatment to improve patient outcomes and quality of life.

## Introduction

 Diabetes mellitus, a metabolic disorder that prevails worldwide, requires the administration of insulin to maintain normal blood glucose levels (BGLs) in patients. Although subcutaneous injections for insulin therapy are available for the management of type 1 diabetes, they have numerous drawbacks, such as tissue damage, anxiety, and pain, which can be challenging for patients, especially children.^1-3^ Therefore, novel approaches to replace hypodermic injection with needle-free high-pressure injection systems or techniques that promote patient compliance are being explored.^4^ Current research is focused on developing micro- and nanoformulations for noninvasive delivery of insulin via inhalation, nasal, percutaneous, and oral routes.^5-12^ Among them, transdermal drug delivery systems (TDDSs) have received immense attention because they are less invasive and more convenient alternatives to subcutaneous insulin injection.^13-15^ Moreover, it has better patient compliance and can deliver insulin across the skin barrier for a longer duration with fewer fluctuations in glucose levels. A reduction in the risk of dose-related side effects results in a better quality of life for diabetic patients. The delivery of insulin through the skin can prevent hepatic first-pass metabolism and degradation through the gastrointestinal tract, which are the significant limiting factors of the oral administration of insulin.^16-18^

 Microneedles (MNs) are a novel and favorable approach for the transdermal delivery of insulin. These are small needles in the micron range in length that are designed to painlessly penetrate the outer layer of the skin and create microscopic channels, allowing for the delivery of drugs through the skin.^19,20^ Compared with traditional injections, MNs can provide more consistent delivery of insulin, which can lead to improved glucose control. In addition, MNs could lead to reduced healthcare costs by eliminating the need for healthcare providers to administer insulin injections.

 Various types of MNs have been developed for insulin delivery, including solid, coated, hollow and dissolving MNs.^21-24^ Each type has its own unique advantages and disadvantages, and continued research to optimize MN design and improve their performance for insulin delivery is in progress.^25,26^ Currently, MNs fabricated using dissolving or biodegradable polymers are explored because they can ensure complete dissolution and degradation within the skin. In addition, these MNs are also nonreusable once removed from a patient’s skin, thus significantly minimizing infection related to transmission. Drugs can be retained within the polymer matrix of MNs, increasing their drug-loading capacity.^27^

 Nanoparticles (NPs) offer a potential solution to the challenges of insulin delivery by encapsulating insulin within a biocompatible and biodegradable material. These NPs protect insulin from degradation and allow for controlled release over an extended duration.^28^

 The present study explored the fabrication of a MN-based insulin delivery system incorporating insulin embedded in polymeric nanoparticles. The MNs were designed to offer the convenience of a lower dose, a reduced frequency of administration, and increased stability of insulin. A pharmacokinetic and pharmacodynamic evaluation of the developed MNs in diabetic rats was performed and is reported in this manuscript.

## Materials and Methods

###  Materials

 Insulin and chitosan (low molecular weight) were purchased from Sigma Aldrich (India), and 4-carboxy phenyl boronic acid and NHS (N-hydroxysuccinimide) were purchased from TCI Chemicals, Chennai, India. 1-(3-Dimethylaminopropyl)3-ethylcarbodiimide hydrochloride (EDC). HCL was procured from Spectrochem Mumbai Pvt. Glacial acetic acid was purchased from NICE Chemicals PVT, Ltd., Kochi, Kerala, India. DMSO was purchased from FINAR Pvt. Sodium hydroxide pellets were purchased from Merck Life Science, Mumbai, India. Polyvinyl alcohol (PVA) was purchased from TCI Chemicals, Chennai, India. Streptozotocin (98%) was purchased from Sigma Aldrich, India. The dialysis membrane (MWCO 12 000 kDa) was purchased from Sigma Aldrich, India. A contour glucometer was purchased from Ascensia Diabetes Care, India. The Rat Insulin ELISA Kit was purchased from Rhetoric Life Sciences Pvt Ltd., India.

###  Methods

####  Synthesis of the chitosan-4-CPBA conjugated polymer

 Chitosan-conjugated 4-carboxy phenylboronic acid (4-CPBA) was synthesized from chitosan and 4-CPBA. Briefly, chitosan (100 mg) was dissolved in 2% glacial acetic acid solution and stirred for 45 min at room temperature to obtain a clear polymeric solution (Solution A). 4-CPBA (112 mg), EDC (175 mg), and NHS (100 mg) were dissolved in DMSO (5 mL) under stirring at room temperature in the dark (Solution B). Solution B was added dropwise to Solution A and stirred for 24 h to form the conjugated polymer. The mixture was dialyzed via a dialysis bag (MWCO 12,000 kDa) against deionized water (5 L) for three cycles to remove unreacted 4-CPBA, crosslinkers, DMSO, and acetic acid.^29^

###  Characterization of the chitosan-4-CPBA conjugated polymer

####  Fourier transform infrared spectroscopy (FTIR)

 FTIR studies were carried out on pure chitosan and 4-CPBA and crosslinked chitosan-4-CPBA via the potassium bromide (KBr) pellet technique. The spectra were recorded via FTIR (FTIR, Shimadzu) within a wavelength range of 400–4000/cm.^30^

####  Differential Scanning Calorimeter (DSC)

 DSC (DSC 60 Plus, Shimadzu) thermograms were obtained for chitosan-conjugated 4-CPBA, 4-CPBA and chitosan. The samples were placed in a sealed aluminum pan, and the heating rate was maintained at 10 °C/min over the temperature range of 25–300 °C under a nitrogen flow rate of 50 mL/min.^31^

####  Powder X-ray diffraction (P-XRD)

 The XRD patterns of chitosan-conjugated 4-CPBA, 4-CPBA, and chitosan were analyzed via a Rigaku Miniflex 600 5^th^ gen. Data were collected in scan mode at a scanning speed of 0.18°/min over a 2theta range of 5–60°. XRD was performed via Cu Kα radiation with a nickel filter, a voltage of 40 kV, and a current of 30 mA.^31^

####  Nuclear magnetic resonance (H1 NMR)

 H1 NMR data of chitosan, 4-CPBA, and 4-CPBA-conjugated chitosan polymers were recorded via a Bruker Ascend 400 MHz. Each sample (3 mg) was transferred into Eppendorf tubes, and 0.5 mL deuterium oxide and 3 µL TFA solution were added and left to dissolve overnight at room temperature for recording via H1 NMR.^29^

####  Development of an HPLC method for the analysis of insulin

 AnHPLC system with a UV detector (Shimadzu) equipped with LC solution software (version 3.1) was used. A YMC-Pack ODS-A column (C18, 250*4.5 mm internal diameter, particle size 5 µm, pore size 12 nm) was used for the separation of insulin. The isocratic mobile phase was composed of 10 mmol anhydrous sodium sulfate (pH 2.3) adjusted with orthophosphoric acid and acetonitrile (70:30). Insulin elution was achieved at a flow rate of 1 mL/min, and the injection volume was 20 μL. The detection was carried out at λ_max_ 214 nm, and insulin was eluted at 12 min. To determine the linearity, standard stock solutions of various concentrations (5, 10, 15, 20, and 25 μg/mL) of insulin were prepared using 0.01 M HCl.^32^

####  Preparation of insulin-loaded chitosan-4-CPBA-conjugated nanoparticles

 The synthesized polymers, chitosan-conjugated 4-CPBA (10 mg) and insulin (10 mg), were dissolved in 0.01 N HCL and mixed under magnetic stirring on a stirrer for 3 h at room temperature. An aqueous solution of 0.5 N NaOH was added dropwise until a white suspension was obtained. A white precipitate was obtained, indicating the formation of NPs.^33^ The formed NPs were separated by centrifugation at 10 000 rpm for 10 min at 4 °C.

####  Characterization of insulin-loaded chitosan-4-CPBA-conjugated nanoparticles

 The mean particle size, zeta potential, and polydispersity index (PDI) of the insulin-loaded chitosan-4-CPBA conjugate NPs were determined via a Zetasizer Nano ZS instrument (Malvern Instruments Limited, UK) at 25 °C.^34^ In addition, the EE and loading capacity (LC) were calculated via the following equations.


$$EE=\frac{Total\;amount\;of\;insulin-Amount\;of\;free\;insulin}{Total\;amount\;of\;insulin}\times 100$$

$$LC=\frac{Total\;amount\;of\;insulin-Amount\;of\;free\;insulin}{Weight\;of\;nanoparticles}\times 100$$

The free insulin was the insulin concentration in the supernatant, which was analyzed after suitable dilution via HPLC. To obtain the weight of the NPs, the supernatant was isolated, and the precipitate was weighed.^35-37^

####  Fabrication of microneedles with insulin-loaded nanoparticles (ILN-MNs)

 The MN master moldconsisted of 225 pyramidal needles (15 × 15 array). The height of the needles was 600 μm, and the space between the two needles was 500 μm. The needles were 200 μm wide at the base. The PVA solution (13%) was prepared by mixing PVA with Milli-Q water under stirring at 90 °C for 4 h. The PVA (13%) stock solution was combined with the ILN-NPs (3:2 ratio). The mixed solution was sealed and kept in a refrigerator (4 °C-8 °C) to remove air bubbles. The ILN-PVA mixture (250 µL) was poured onto the surface of the PDMS micromold, and the molds containing the formulation were kept in a rotary shaker for approximately 30 min to enable the penetration of the ILN-PVA mixture into the cavities of the MN. The mold was left overnight at 18 °C for drying.^38,39^ The fabricated MNs were stored in a desiccator until further use. For the preparation of bank MNs, a 13% PVA solution was poured into the mold in a similar manner to form blank MNs (blank MNs).

###  Characterization of the MNs

####  Scanning electron microscopy 

 The surface morphology and microneedle dimensions were analyzed via SEM (Hitachi tabletop, TM3030Plus). The optimized blank-MNs and ILN-MNs arrays were mounted on a sample stub with two-sided adhesive tape and observed under magnifications of 300 X and 100 X with an accelerating voltage of 5 kV.^40^

####  Measurement of the mechanical failure force of the MNs

 The mechanical properties of the prepared MNs were studied via TA. XT plus texture analyzer (Stable Micro Systems, Surrey, UK). The MN arrays (blank MNs and ILN-MNs) with the needle tips facing upward were placed on a flat rigid aluminum plate. Using a 5-mm-diameter flat-head stainless steel cylindrical probe, an axial force oriented perpendicularly to the plate (i.e., parallel to the MN vertical axis) was applied. The probe was able to press against the tips of the MN array at a constant speed of 1.1 mm/sec, and the trigger force was set at 50 N. The force required to move the metal cylinder as a function of distance until a displacement of 400 μm was reached was recorded by the texture analyzer.^41,42^

####  Parafilm insertion study

 A sheet of parafilm (Parafilm M^®^, a blend of hydrocarbon wax and a polyolefin) was folded upon each other to obtain an eight-layered model membrane simulating skin tissue (~1 mm thickness). The test was performed via the manual insertion method, where the MN patch was placed over the parafilm sheet and held for 30 s with a downward force using a thumb impression. The MN patch was then removed from the parafilm membrane after insertion. Each layer was subsequently separated and visually inspected for the formation of holes in every layer to measure the depth to which the MNs could penetrate.^43^

####  Microchannel depth by confocal laser scanning microscopy

 The depth of insertion of the developed ILN-MN patch was examined via confocal laser scanning microscopy on freshly excised rat skin. The skin was treated with the ILN-MN patch via a thumb press and held in place for 1 h using occlusive tape. The porated site was treated with 50 μL of Rhodamine B, a fluorescent dye, for 30 min, after which the site was blotted with wet tissue wipes to remove excess dye. The tissue was then mounted on a coverslip and examined via a Leica SP-DMi8 laser scanning confocal microscope at 20 × magnification. Excitation was carried out at a wavelength of 543 nm, and emission was carried out at 569 nm via an argon laser. XYZ sectioning was performed to detect the depth of the fluorescent dye penetration. The optical sections were reconstructed into a 3D model via Leica application suite X software. The depth of the microchannels was inferred indirectly on the basis of the migration of Rhodamine B down the microchannel.^44,45^

####  Quantification of insulin in the MNs

 To determine the content of insulin loaded in the ILN-MNs, insulin was extracted from the MNs with 1.5 mL of 0.01 N HCL followed by vortex mixing to release the encapsulated insulin completely. The supernatant obtained after centrifugation for 10 min was separated and analyzed via the developed HPLC method.

###  In vivo evaluation of the MNs in diabetic rats

####  Induction of diabetes

 Animal studies were conducted in accordance with approval from the Institutional Animal Ethics Committee, Kasturba Medical College, Manipal Academy of Higher Education, Manipal (Approval number: IAEC/KMC/20/2019). Male Wistar rats weighing 250 ± 20 g were used. Before the experiment, the animals were acclimatized in laboratory settings for 7 days and housed at 20–25 °C with 75% humidity and a 12:12-h light:dark cycle with unlimited food and water. For the induction of diabetes, the rats were fasted for 12 hr. with free access to water. Diabetes was induced by a single intraperitoneal injection of 40 mg/kg STZ dissolved in normal saline.^46^

####  Pharmacokinetic and pharmacodynamic evaluation

 The study commenced on the 8th day following STZ administration. Diabetic rats were fasted for 8 h prior to the experiment and remained fasted for 12 h during the experiment but had free access to water. The rats were divided into 4 groups (N = 4). Group 1 served as a positive control, group 2 received blank-MNs, group 3 was administered plain insulin (10 IU) via subcutaneous injection, and group 4 received ILN-MNs. The method of blood collection was randomized. Blood was collected alternatively from each animal in their respective group at different time points during the 12-hr study. Blood was collected from each rat at four time points to avoid pain and stress.^47^

 For subcutaneous injection, plain insulin was dissolved in 0.01 N HCl and mixed in a normal saline (1:9) ratio. In the case of transdermal application of the MNs, the fur of diabetic rats at the dorsal area was shaved. Blood samples (0.2 mL) were collected at various time points (0.5, 1, 2, 4, 6, 8, 10, and 12 h) through the retro-orbital plexus.^48^ Before blood samples were withdrawn, the rats were anesthetized with diethyl ether. The blood samples were allowed to stand for 1 h and then centrifuged at 6000 rpm and 4 °C for 10 min to isolate the serum, which was subsequently stored at -20 °C until further analysis. The BGLs were measured via a contour glucometer. A rat insulin ELISA kit was used to estimate the serum insulin levels at a wavelength of 450 nm (Rhetoric Life Science, India). Phoenix WinNonlin software was used for the estimation of the PK parameters.^49^

###  Statistical analysis

 The results are expressed as the means ± SEMs. All the statistical calculations were performed via GraphPad Prism software (version 8.0.2.) for Windows, GraphPad Software (San Diego, California, USA).

## Results and Discussion

###  Characterization of the chitosan-4-CPBA conjugated polymer

####  Fourier transform infrared spectroscopy 

**Figure 1:**
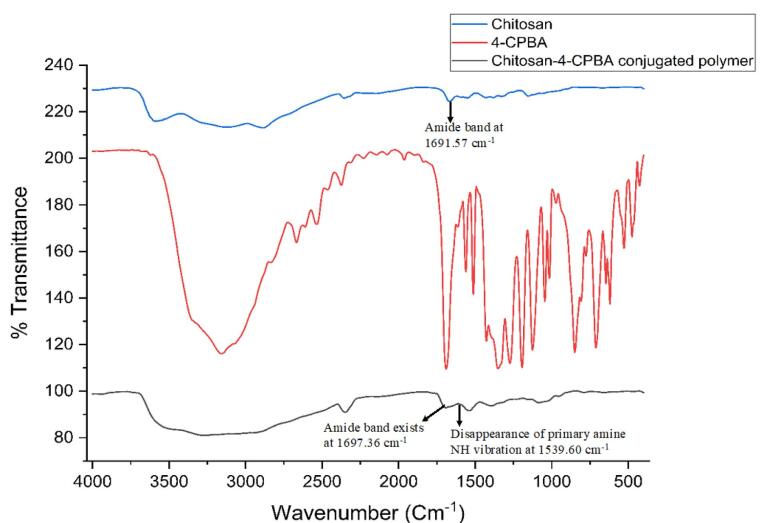
FTIR spectra of chitosan, 4-CPBA and the chitosan-4-CPBA-conjugated polymer

 The FTIR spectra of chitosan, 4-CPBA, and the chitosan-4-CPBA conjugated polymer are depicted in Figure 1. The pure chitosan exhibited a primary amine peak at 1598.99 cm^-1^; free amine stretching at 3400-3500 cm^-1^, amide-linked carbonyl groups at 1691.57 cm^-1^; and bending from saturated hydrocarbons at 1431 cm^-1^, 1379.10 cm^-1^, and 1327.03 cm^-1^; and C-O stretching at 1064.71 cm^-1^, which is in accordance with the chitosan FTIR spectra reported in the literature.^50,51^ The FTIR spectrum of 4-CPBA exhibited a characteristic C = O unsaturated aromatic ring acid present at 1689.64 cm^-1^, a boronic acid group (O-B-O) present at 1327.03 cm^-1^, and di-substituted benzene rings at 646.15 cm^-1^ and 848.68 cm^-1^, which agrees with the spectra reported in the literature.^51^ The formation of the chitosan-4-CPBA conjugate was confirmed by the existence of an amide band at 1697.36 cm^-1^ in the FTIR spectrum of the chitosan conjugate polymer. The characteristic peak of the primary amine NH vibration appeared at 1598.99 cm^-1^ in the spectrum of chitosan and disappeared in the spectrum of the chitosan-CPBA conjugated polymer. Characteristic absorption bands of the para-substituted benzene ring were observed at 846.75 cm^-1^, 653.87 cm^-1^, and 460.99 cm^-1^ for the chitosan-4-CPBA-conjugated polymer, which is in accordance with the reported literature.

####  Differential scanning calorimetry 

**Figure 2:**
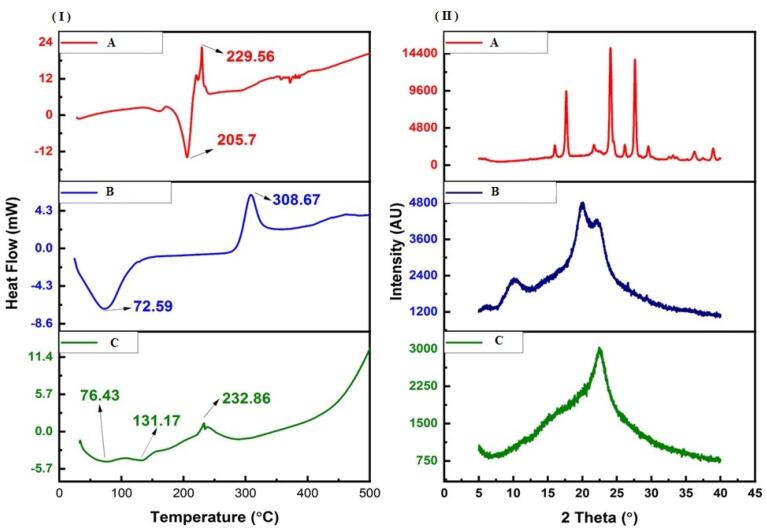
DSC thermograms (I) and P-XRD diffractograms (II) of A: 4-CPBA, B: chitosan and C: chitosan-conjugated 4-CPBA polymers

 The change in the material attributes of chitosan after conjugation with 4-CPBA during the formation of the chitosan-4-CPBA conjugate polymer was studied via DSC analysis (Figure 2 (I)). Pure 4-CPBA exhibited an endothermic peak at ~205 °C, corresponding to its melting point. Notably, phenylboronic acids usually exhibit inconsistent melting points; hence, the endothermic peaks are considered decomposition temperatures. The peak is also attributed to the initial dehydration reaction of the phenylboronic acid forming the anhydrides.^52^ Additionally, an exothermic peak was observed at ~229 °C, indicating a possible solid‒solid transformation, which has been previously reported. On the other hand, pure chitosan exhibited two prominent peaks, namely, an endothermic peak at ~72 °C and an exothermic peak at ~308 °C. The endothermic peak is attributed to the release of water molecules from the -NH_2_ and -OH groups present in the chitosan chains, and the exothermic peak is attributed to the degradation and depolymerization of the chitosan polymer.

 After the formation of the chitosan-4-CPBA conjugate polymer, the DSC thermograms presented two endothermic peaks at ~76 °C and ~131 °C and an exothermic peak at ~232 °C. The peak at ~76 °C corresponds to the endothermic peak of chitosan; however, it shifted slightly toward higher temperatures. The additional peak at ~131 °C may be attributed to the continued dehydration of the chitosan-4-CPBA conjugate polymer owing to the presence of hydrophilic groups. The exothermic peak at ~232 °C corresponds to the degradation and depolymerization of the polymeric chain, as in pure chitosan (occurring at ~308 °C). The lower shift in the degradation temperature for the chitosan-4-CPBA conjugate polymer can be correlated with the loss of free -NH_2_ and -OH groups due to conjugation with 4-CPBA.^53,54^ The absence of the peak at approximately 205 °C (melting point of 4-CPBA) clearly demonstrated the complete removal of the unreacted 4-CPBA by dialysis.

####  Powder X-ray diffraction 

 P-XRD was carried out to determine the physical changes in the material attributes of the 4-CPBA, chitosan and chitosan-4-CPBA conjugate polymers (Figure 2(II)). 4-CPBA is a crystalline material^55^ and presents characteristic XRD peaks at 16°, 17.66°, 21.7°, 24.08°, 26.14°, 27.62°, 29.54°, 36.18°, and 38.92°. Chitosan, a polymer, exhibited two characteristic peaks at 10.24° and 19.92°, which is consistent with previously reported values corresponding to its semicrystalline nature.^56^ Chitosan also presented two additional peaks at 6.28° and 22.28°. The overall crystallinity index of 4-CPBA was 82.41%, and that of chitosan was 58.15%. However, the chitosan-4-CPBA conjugate polymer only exhibited one peak at 22.56° and a crystallinity index of 26.63%. The chitosan-4-CPBA conjugate polymer was clearly amorphous in nature, and the peak of chitosan at ~19° shifted to a higher angle (~22°), which can be attributed to successful conjugation with 4-CPBA. Furthermore, the absence of a peak at < 12° confirmed the change in the material attributes of the chitosan-4-CPBA conjugate polymer compared with those of the pristine 4-CPBA and chitosan. The observed shift in the peak of the chitosan-4-CPBA conjugate polymer is in good agreement with previous observations with conjugated chitosan.^57-59^

####  Nuclear Magnetic Resonance (H1 NMR)

**Figure 3:**
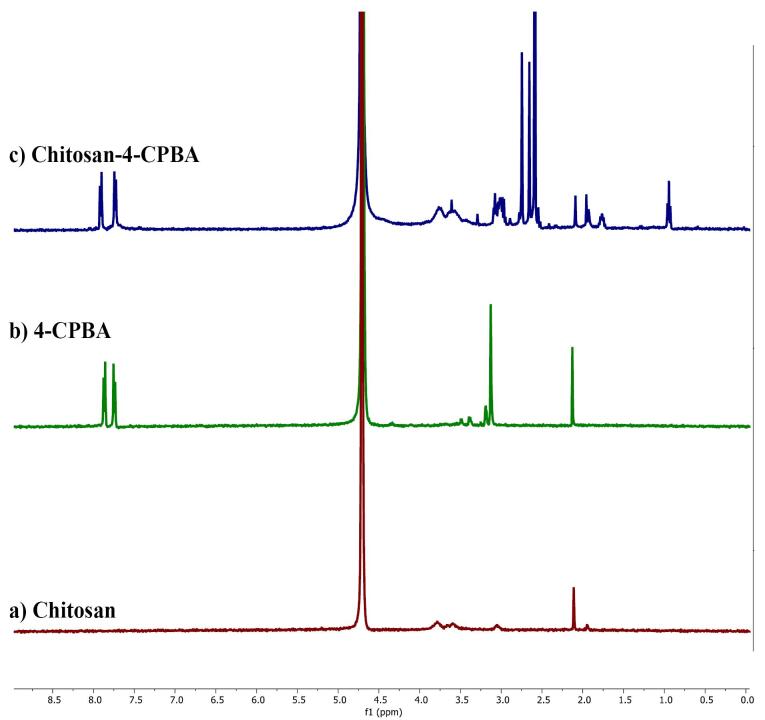
H1 NMR spectra of a) chitosan, b) 4-CPBA, and c) chitosan-4-CPBA

 The NMR spectra (Figure 3) of chitosan exhibited the characteristic peaks of chitosan, which were visible at 2.1 ppm. The H1 NMR spectrum of 4-CPBA had peaks at 2.1, 3.1, 7.7, and 7.8 ppm. The H1 NMR spectrum of the chitosan-4-CPBA conjugate polymer showed peaks at 2.5 ppm to 2.7 ppm, 7.7 ppm, and 7.9 ppm. The distinctive peaks observed in the spectra of chitosan-conjugated 4-carboxy phenyl boronic acid were found at 2.0 ppm (-CH3 from the acetylated segment of chitosan) and 3.1-3.8 ppm (protons from the glucosamine ring). Additional peaks at 7.8-8.0 ppm, corresponding to the phenyl ring protons from the boronate moiety, were observed, indicating that the phenyl boronate groups were successfully conjugated to chitosan. The spectral data are in good agreement with those reported in the literature.^60,61^

####  Development of an HPLC method for the analysis of insulin

 TheHPLC method for the estimation of insulin in the developed formulation was adopted from the previously reported method by Rajan et al, with certain modifications.^32^ The linearity graph was obtained by plotting different concentrations of insulin (x-axis) versus the average peak area (y-axis). A linear relationship was observed at concentrations ranging from 5–25 μg/mL. y = 17404x–18600 was found to be a linear regression equation with a correlation coefficient of r^2^ = 0.9997. The limit of detection (LOD) and limit of quantification (LOQ) were 0.70 and 0.65 μg/mL, respectively.

####  Insulin-loaded chitosan-4-CPBA-conjugated nanoparticles: Particle size, zeta potential, drug encapsulation efficiency and loading capacity

**Figure 4:**
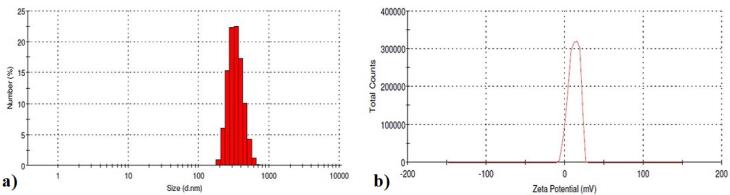
a) Particle size and b) zeta potential of insulin-loaded chitosan-4-CPBA-conjugated nanoparticles

 The insulin-loaded chitosan-4-CPBA nanoparticles had an average particle size of 509.34 ± 31.14 nm, a zeta potential of 11.7 ± 6.8 mV and a polydispersity index of 0.25 ± 0 (Figure 4a and 4b). The results were consistent, which indicates that the uniform size of the NPs was maintained during the NP formulation process.

 The EE describes the system’s ability to entrap the therapeutic moiety within the nanocarriers, whereas the LC represents the amount of therapeutic moiety loaded per unit weight of the nanoparticle.^62^ Both of these parameters are crucial for the evaluation of nanoparticles. The EEwas determined after separation of the NPs by centrifugation. To calculate the EE, the amount of free insulin in the supernatant was determined. The insulin-loaded chitosan-4-CPBA-conjugated NPs had an EE of 80%, and the loading capacity (LC) was 30%.

 Zhang et al^36^ prepared insulin-loaded PEG-g-chitosan nanoparticles by ionotropic gelation. The reported EE of insulin-loaded NPs was > 78%, and the LC was 38.6%. Similar findings were reported by Wu et al^63^ in a study involving insulin-loaded phenyl boronic acid-grafted chitosan nanoparticles (PBACSNPs) for controlled insulin release. The insulin-loaded PBACS NPs displayed a high EE of 53.6% and an LC of 17.9%, with sizes ranging from 500-700 nm and zeta potentials ranging from + 2.7-7.21 mV. Siddiqui et al^64^ prepared 4-formylphenylboronic acid-conjugated chitosan and used this conjugate to formulate insulin-containing NPs via polyelectrolyte complexation. The NPs had an EE of 81% and an insulin LC of 46%, and these NPs had a z-average of 140 ± 12.8 nm, a zeta potential of + 19.1 ± 0.69 mV and a PDI of 0.17 ± 0.1. The EE, LC, zeta size, zeta potential and PDI of our NPs are in accordance with the results of studies reported for insulin-loaded NPs in the literature.

####  Scanning electron microscopy 

**Figure 5:**
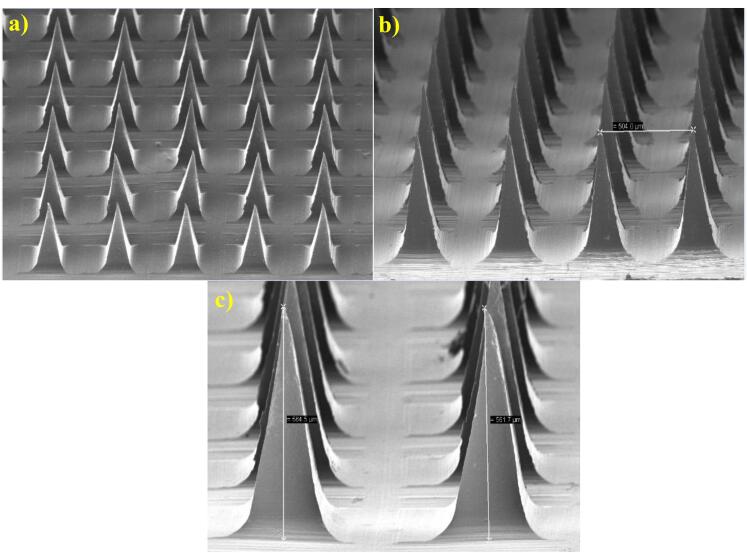
SEM images of microneedles under magnifications of 300 × and 100 × with an accelerating voltage of 5 kV. a) Magnifications of 100 × , b) magnification of 150 × , and c) magnification of 300 ×

 SEM is a powerful tool used for characterizing the morphology and surface topography of MNs. SEM can provide high-resolution images of the MN surface, which can be used to evaluate the size and shape of the MNs. The SEM images of insulin-loaded MNs typically show an array of micron-sized needles with sharp tips and uniform dimensions (Figure 5). A typical digital image of the MNs indicates successful replication of the mold. The SEM image of the MNs revealed a pyramid-shaped shape with a height of approximately 560 μm. The distance of the needle tip between two MNs was ~504 μm, and the width of the base for each MN was ~283 μm. The MNs were long and sharp.

####  Measurement of the mechanical failure force of the MNs

 The measurement of the mechanical failure force is an important parameter for evaluating the effectiveness and safety of MNs for transdermal drug delivery. Polymer MNs should have sufficient mechanical strength for penetrating the skin without mechanical failure. The mechanical failure force refers to the force required to break or fracture a microneedle and is an indicator of the strength and durability of the MN.

**Figure 6:**
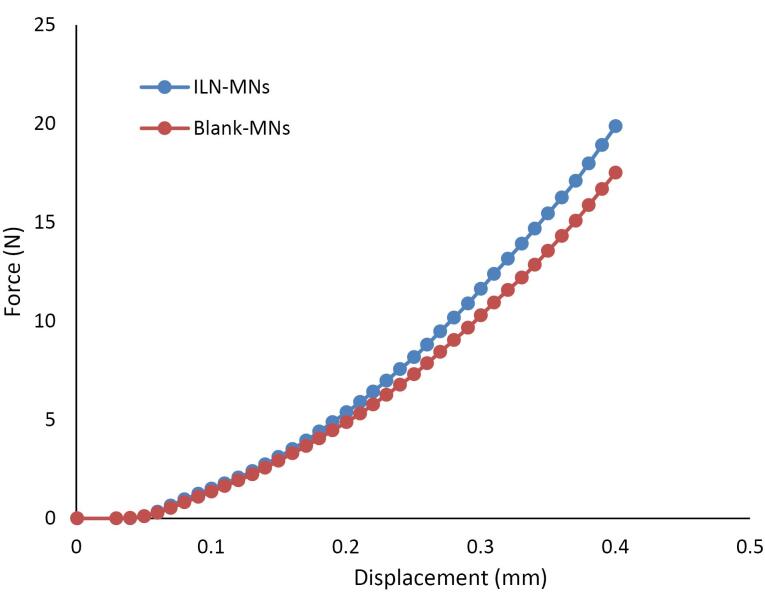
Mechanical failure force analysis of microneedles

 The mechanical characteristics of the prepared dissolving MN patches were evaluated via the TA-XT texture analyzer compression mode, which continuously recorded the compression force and displacement. The mechanical strengths of the blank-MNs and ILN-MNs (Figure 6) indicate that the force required to press the metal cylinder against the needle increased with displacement over the tested range. The absence of discontinuous points in the force‒displacement curves confirmed the integrity of the needle. For the blank-MNs, the compression force reached 18 N when the displacement reached 400 μm, whereas for the ILN-MNs, the force reached 22 N when the displacement reached 400 μm. According to reported studies, fabricated MNs have sufficient strength to penetrate the skin.

####  Parafilm insertion test

**Figure 7:**
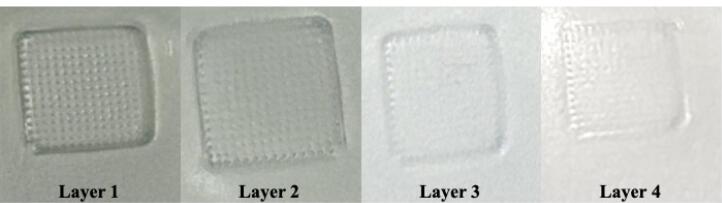
Parafilm insertion test

 The parafilm insertion test provides an indication of MN penetration as a function of depth. The fabricated MNs displayed visible holes in the first and second layers of parafilm, indicating complete penetration. However, the holes in the third and fourth layers of the parafilm were not clearly visible, indicating partial indentation (Figure 7). The thickness of the stratum corneum ranges between 2 and 20 μm at different sites, and that of the epidermis is approximately 80 μm. Considering that the average thickness of each parafilm layer was approximately 127 μm,^65^ the developed MNs were able to penetrate to a depth of 254 μm. The results obtained indicate that the MNs could easily breach the stratum corneum and reach beyond the epidermis.

####  Microchannel depth by confocal laser scanning microscopy

**Figure 8:**
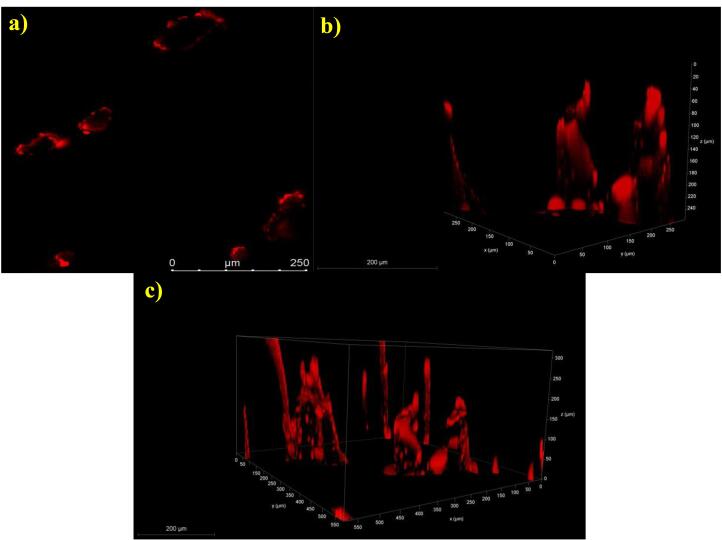
Confocal laser scanning microscopy images of the microchannels created upon application of the ILN-MNs: (a) pores created by the ILN-MN patch on the skin and (b) and (c) 3D images of the ILN-MN-treated rat skin illustrating the insertion depth

 A rat skin sample was imaged and recorded to determine the depth at which the ILN-MN patch was inserted from the surface of the skin via confocal laser scanning microscopy, as shown in Figure 8. The penetration of Rhodamine B dye indicates the depth of the created microchannel, which resulted in a mean depth of 242.5 ± 28.72 μm (n = 4). These findings are correlated with the insertion depth as determined by in vitro experiments (parafilm insertion study). The modest variation may be attributed to certain factors that directly affect the depth of the microchannels, such as the force and time of application, moisture content and elasticity of the skin. Figure 8a shows a representative image of the pores created upon application of the ILN-MN patch on the skin, whereas 8b and 8c depict the microchannels created by the ILN-MN patch. Hair follicles have also been observed.

####  In vivo pharmacokinetic and pharmacodynamic evaluation of the MNs

 The in vivo pharmacokinetics and pharmacodynamics of the MNs were studied in 4 groups of diabetic rats. While the first group served as a positive control, the second group received blank-MNs that were devoid of insulin, the third group received plain insulin (10 IU) via subcutaneous injection, and the fourth group received the fabricated MNs incorporated with the insulin nanoparticles (ILN-MNs, 20 IU). These groups were included in the study to compare the performance of the MNs with that of the subcutaneous injection of plain insulin.

**Figure 9:**
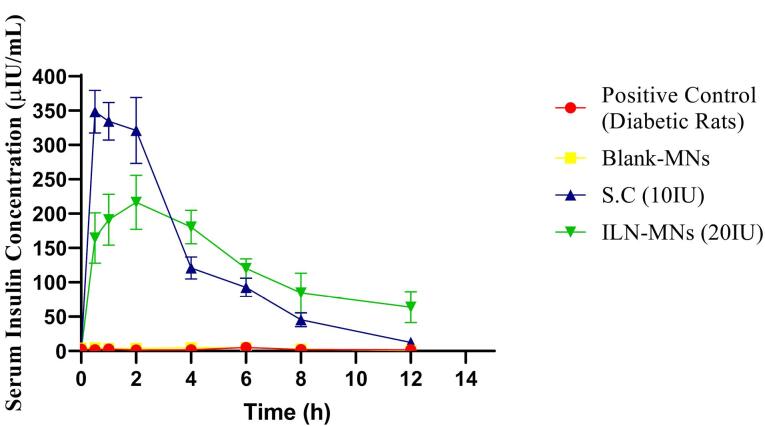
Serum insulin concentrations of diabetic rats from different treatment groups (SC: subcutaneous insulin injection; ILN-MNs: microneedles incorporated with insulin-loaded nanoparticles; IU: international units)

**Figure 10:**
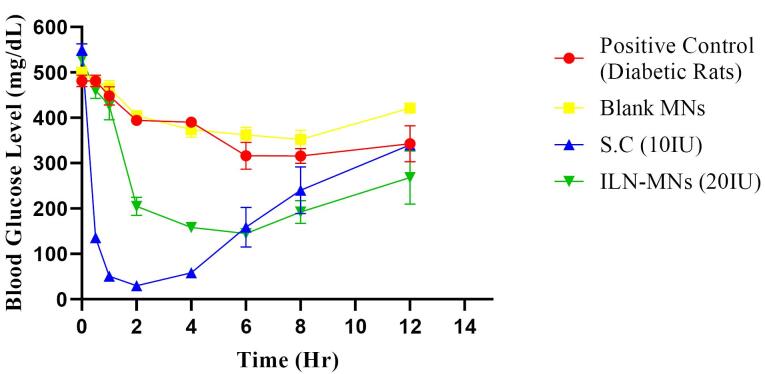
The blood glucose levels of diabetic rats from different treatment groups (S. C: Subcutaneous insulin injection, ILN-MNs: Microneedles incorporated with insulin-loaded nanoparticles, IU: International Units)

 The serum insulin concentration was estimated via a rat insulin ELISA kit, the results of which are displayed in Figure 9. The serum insulin concentration reached a maximum (C_max_) of 348.34 µIU/mL at 0.5 h, and the insulin levels remained stable for 2 h following the subcutaneous administration of insulin (10 IU). Thereafter, it decreased to 120 µIU/mL in the fourth hour, and a steep decline (45 µIU/mL at 8 h) was observed. The BGLs of diabetic rats after various treatments are shown in Figure 10. The positive control group and blank-MNs group did not exhibit hypoglycemic effects, and their BGLs remained elevated during the study period. Hypoglycemia was observed in diabetic rats that were administered plain insulin (10 IU) subcutaneously. The BGL in this group decreased to 200 mg/dL at 0.5 h following insulin administration and decreased further to its lowest level of 29 mg/dL at 2 h. At 6 h, the BGL increased to its initial level. In diabetic rats treated with ILN-MNs (20 IU), the BGLs reached ≤ 200 mg/dL at 2 h following the application of the MNs, and the BGLs were maintained at ≤ 200 mg/dL from 2-8 h.

 The AUC_0-12_ obtained for subcutaneous injection (10 IU) was 1495.35 h*µIU/mL. The maximum serum insulin concentration (C_max_) observed in the rats treated with the ILN-MNs (20 IU) was 216 µIU/mL at 2 h following the application of the MNs, and the AUC_0-12_ obtained was 1533.67 (h*µIU/mL) (Table 1). Data are expressed as the mean ± SEM and were analyzed via one-way ANOVA followed by post hoc Tukey’s multiple comparison tests. There was no statistically significant difference in the serum insulin concentration following the administration of subcutaneous insulin injection compared with insulin nanoparticle-loaded MNs (ILN-MNs). Additionally, there was no statistically significant difference in the number of BGLs with subcutaneous insulin injection compared with the number of ILN-MNs.

**Table 1:** Pharmacokinetic parameters of insulin from ILN-MNs patches and S.C. insulin administration in diabetic rats (n = 4)

**Group**	**Dose (IU)**	**C max (µIU/mL)**	**T max (h)**	AUC last (h*µIU/mL)
S.C	10	348.35 ± 1.4	0.5 ± 0.08	1495.35 ± 1.1
ILN MN	20	216.66 ± 1.1	2 ± 0.18	1533.67 ± 1.7

SC: subcutaneous insulin injection; ILN-MNs: microneedles incorporating insulin-loaded nanoparticles; IU: international units.

 These findings show that MNs exhibit a longer and more sustained release profile of insulin than does subcutaneous injection. Delivery of insulin can be challenging owing to its susceptibility to enzymatic degradation and rapid clearance from the bloodstream. Anbazhagan and Suseela developed insulin-coated MNs and evaluated their BGLs in diabetic rats.^66^ The formulation developed in the current study had insulin loaded inside the MNs, which ensured the protection of insulin, unlike the reported formulation in which insulin was coated on the MN surface. Additionally, in our study, we evaluated BGLs and serum insulin concentrations. Most insulin-MN studies only measure BGLs, and the corresponding insulin concentration is not reported. The nanoparticles protect insulin from degradation and allow for controlled release over an extended period.^67^ Transdermal delivery of insulin via MNs aids in sustaining insulin release, thereby preventing the rapid decrease in BGLs and minimizing hypoglycemia-related adverse effects.

## Conclusion

 In this study, MNs loaded with insulin nanoparticles were fabricated to overcome the drawbacks of traditional insulin injections. Insulin was loaded into nanoparticles prepared with a chitosan-4-carboxyphenyboronic acid-conjugated polymer. The prepared insulin-loaded nanoparticles were loaded in PVA-based MNs. The fabricated MNs exhibited sufficient mechanical strength to penetrate the skin. The in vivo efficacy of insulin nanoparticle-loaded MNs was studied in diabetic Wistar rats in comparison with that of subcutaneous insulin injection. The BGLs and the insulin concentration demonstrated the superiority of the insulin NP-loaded MNs over traditional subcutaneous insulin injection. Overall, the fabricated MNs exhibited convincing efficacy as a promising approach for transdermal delivery of insulin, offering potential advantages over traditional injection methods. Further research is needed to optimize the MN design and evaluate its safety and efficacy for long-term use.

## Competing Interests

 The authors have no conflicts of interest to declare.

## Ethical Approval

 The current research work was approved by the Institutional Animal Ethics Committee (Registration no. 94/PO/ReBi/S/99/CPCSEA), Kasturba Medical College, Manipal Academy of Higher Education, Manipal. The ethical approval code for this study is IAEC/KMC/20/2019.
